# Current understanding of extrachromosomal circular DNA in cancer pathogenesis and therapeutic resistance

**DOI:** 10.1186/s13045-020-00960-9

**Published:** 2020-09-14

**Authors:** Yuanliang Yan, Guijie Guo, Jinzhou Huang, Ming Gao, Qian Zhu, Shuangshuang Zeng, Zhicheng Gong, Zhijie Xu

**Affiliations:** 1 Department of Pharmacy, Xiangya Hospital, Central South University, Changsha, 410008 Hunan China; 2 Department of Oncology, Mayo Clinic, Rochester, MN 55905 USA; 3 Department of Pathology, Xiangya Hospital, Central South University, Changsha, 410008 Hunan China; 4 National Clinical Research Center for Geriatric Disorders, Xiangya Hospital, Central South University, Changsha, 410008 Hunan China

**Keywords:** Extrachromosomal circular DNA, Oncogene amplification, Therapeutic resistance, Cancer pathogenesis, Biomarkers, Clinical utility

## Abstract

Extrachromosomal circular DNA was recently found to be particularly abundant in multiple human cancer cells, although its frequency varies among different tumor types. Elevated levels of extrachromosomal circular DNA have been considered an effective biomarker of cancer pathogenesis. Multiple reports have demonstrated that the amplification of oncogenes and therapeutic resistance genes located on extrachromosomal DNA is a frequent event that drives intratumoral genetic heterogeneity and provides a potential evolutionary advantage. This review highlights the current understanding of the extrachromosomal circular DNA present in the tissues and circulation of patients with advanced cancers and provides a detailed discussion of their substantial roles in tumor regulation. Confirming the presence of cancer-related extrachromosomal circular DNA would provide a putative testing strategy for the precision diagnosis and treatment of human malignancies in clinical practice.

## Introduction

**Fig. 1:**
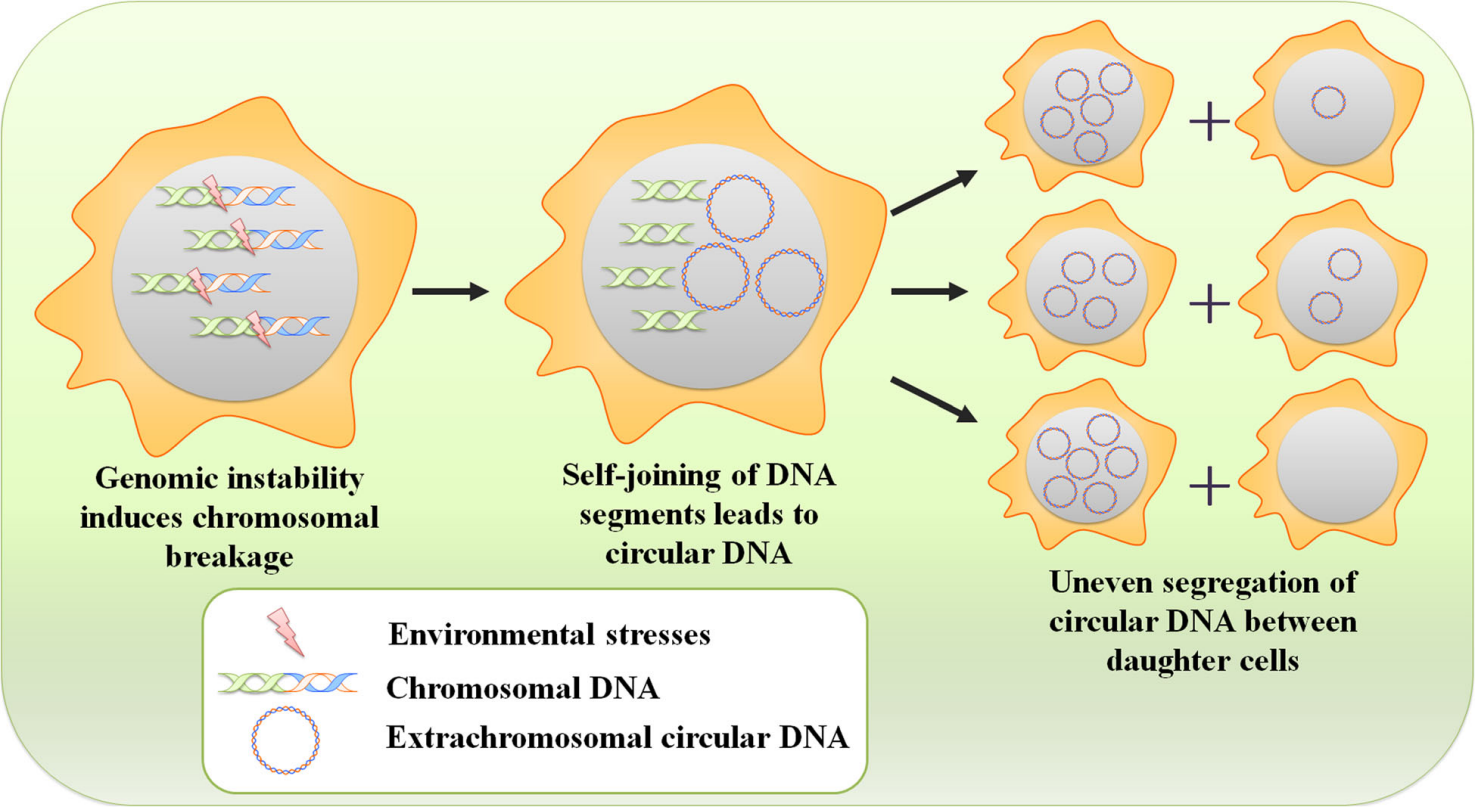
Inheritance of extrachromosomal circular DNA elements. Environmental stresses cause genomic instability and drive local chromosomal breakage. After then, the self-joining of DNA segments leads to formation of extrachromosomal circular DNA. Because of lacking centromeres, extrachromosomal circular DNA elements can be inherited unequally between daughter cells, contributing to intratumoral heterogeneity and cancer cell progression

Extrachromosomal circular DNA, first described by Hotta’s group in 1964, is highly conserved across multiple species [1]. Other research groups further identified the existence of extrachromosomal circular DNA in cells by karyotype preparations [2] or by chloride density gradients [3]. These circular DNA elements carry sequences that are homologous to genomic DNA [4], but are distinct from mitochondrial DNA [5] and viral covalently closed circular DNA [6]. Since the discovery of extrachromosomal circular DNA, biomedical research has led to the general view that tumor-associated extrachromosomal circular DNA has an adverse effect on human health and accelerates malignant behaviors [7]. These circular DNA molecules that exist in cancer cells can be divided into at least two classes based on their different sizes and copy numbers: (i) small—usually less than 10 Kb, termed eccDNA, e.g., microDNA [8]; and (ii) large–often greater than 1 Mb, termed ecDNA, e.g., double minutes (DMs) [9]. Numerous studies have been conducted to investigate the relationship between extrachromosomal circular DNA and cancer biology, and these DNA elements may serve as promising biomarkers for cancer research and treatment. An early example of the importance of extrachromosomal circular DNA elements in tumorigenesis was the discovery of DM structures in malignant tumors from children [10]. Using a combination of whole-genome sequencing (WGS), cytogenetic analyses and structural modeling, Turner’s group identified that extrachromosomal circular DNA is common and highly amplified in many types of cancers, but these molecules can vary greatly among different cells from a single individual [11], confirming previous studies [12–14]. Of particular interest in this context is that most extrachromosomal circular DNA can amplify genes, including oncogenes in cancers, and can influence gene expression profiles, contributing to oncogenesis [15, 16]. The oncogenes amplified in circular DNA structures were shown to lead to high levels of mRNA transcripts, such as *epidermal growth factor receptor (EGFR)*, *mouse double minute 2 (MDM2)*, and *cyclin D1 (CCND1)* [11, 16]. In addition, circle-derived genomic rearrangements further contribute to the aberrant expression of cancer-relevant genes, such as *doublecortin-like kinase 1 (DCLK1)* and *telomerase reverse transcriptase (TERT)* [17]. Furthermore, unlike the circular neochromosomes [18] and ring chromosomes [19] that contain centromeres, studies have found that the eccDNA/ecDNA elements, in the context of this review, lack centromeres, resulting in their uneven segregation from parental cells to daughter cells during cell division (Fig. 1); this uneven segregation accelerates cancer progression in changing environments [20, 21]. The characteristics of circular DNA show its primary effects on the expression of cancer-relevant genes in tumorigenesis, indicating that it is a powerful driver of intratumoral heterogeneity and progression, independent of chromosomal DNA alterations [22].

**Fig. 2:**
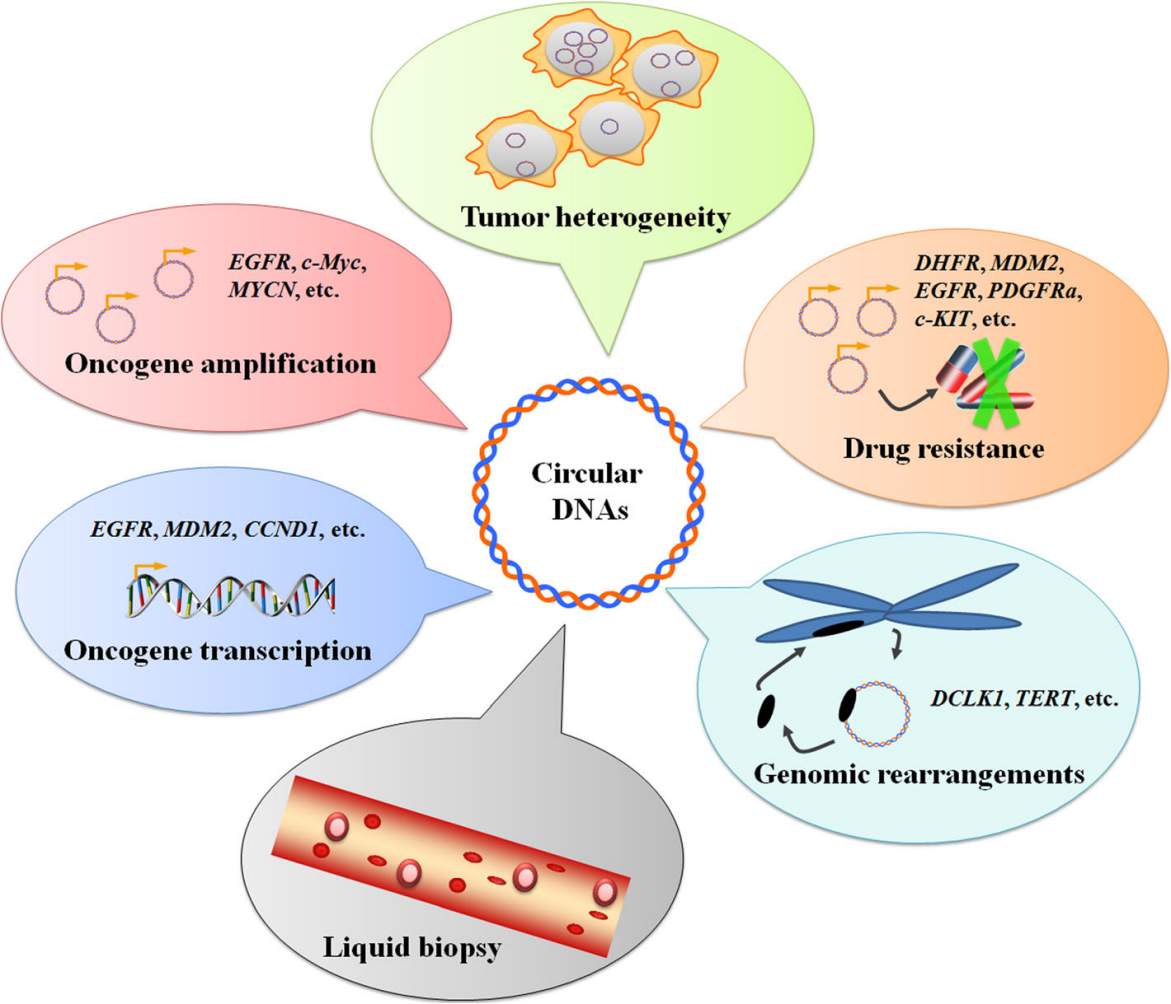
Current understanding of extrachromosomal circular DNA in cancer pathogenesis. Multiple recent studies have demonstrated the increasing importance of extrachromosomal circular DNA on oncogenic features and regulatory capacity in cancer research and treatment

In this review, we focused exclusively on extrachromosomal circular DNA and its substantial tumor-regulating roles in cancer pathogenesis (Fig. 2). DMs, microDNA, and other types are discussed, highlighting their major contribution to tumor pathogenesis and treatment response. Evaluating the potential mechanisms and functions of these molecules will enable a better understanding of tumorigenesis in an effort to provide fresh and novel insights for developing biomarker applications and drug–drug combinations to improve anticancer outcomes.

### Biogenesis of extrachromosomal circular DNA in cancer

**Fig. 3:**
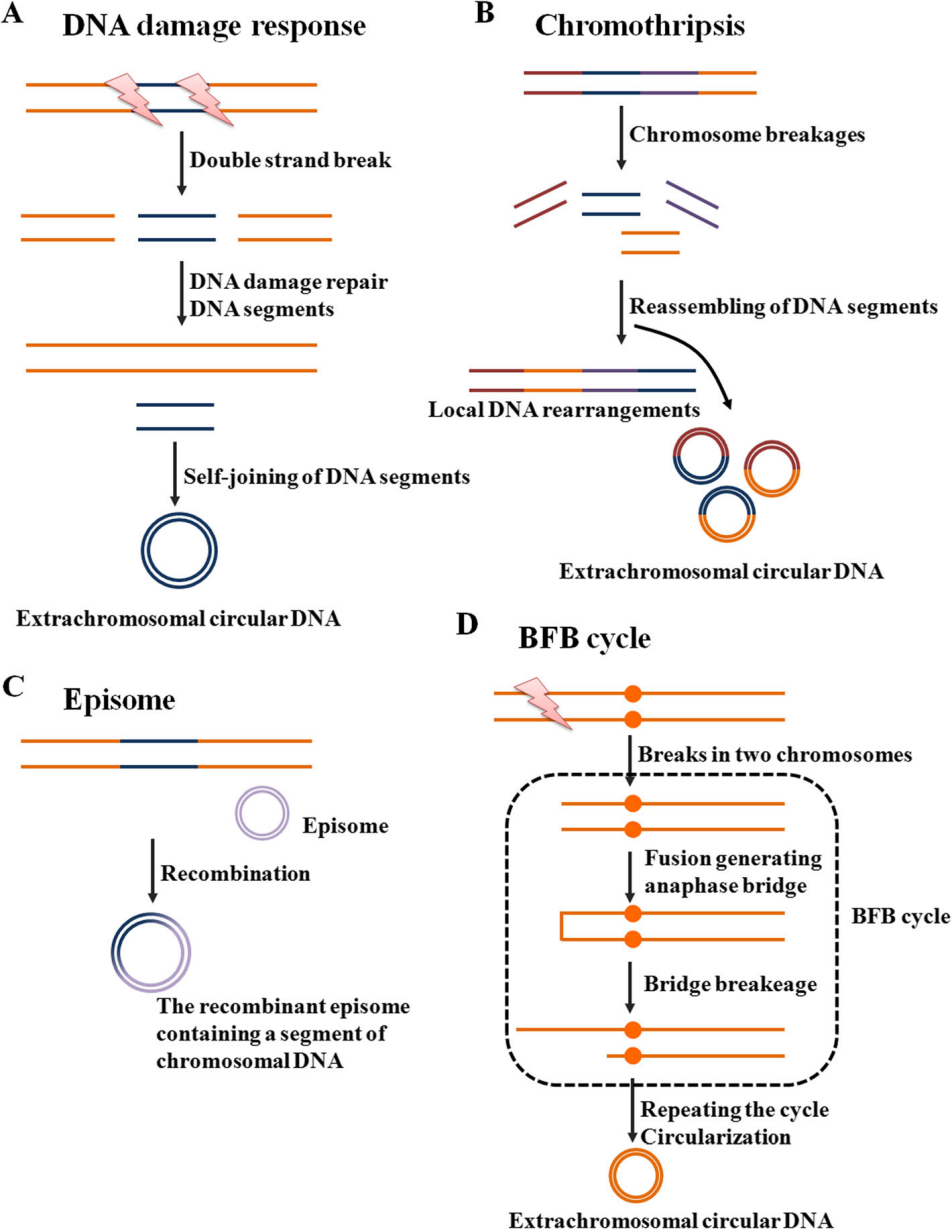
Models of how extrachromosomal circular DNA is formed. **a** In response to environmental changes, cells could repair DNA strand break through removing the damaged segments. After then, the small DNA segments could form circular DNA. **b** Chromothripsis is a single-step catastrophic event that drives chromosome breakage and end-to-end chromosomal fusion, and serves as the underlying driving force linked to local DNA rearrangements and extrachromosomal circular DNA. **c** The episomes are autonomously replicating submicroscopic precursors of extrachromosomal circular DNA. In cell, The DNA recombination processes can generate the recombinant episome that contains a segment of chromosomal DNA, such as *c-Myc*-containing DMs. **d** The BFB cycle involves anaphase bridge formation due to the presence of dicentric chromosomes, followed by bridge breakage, which generates a variety of chromosomal aberrations, including extrachromosomal circular DNA

The underlying molecular mechanisms that lead to the biogenesis of extrachromosomal circular DNA have yet to be fully clarified. Both DNA damage and the corresponding DNA repair strategies, the important events of genomic homeostasis observed particularly in tumorigenesis [23–26], are processes that have been proven to produce extrachromosomal circular DNA species [27–29] (Fig. 3). The microDNA is often observed from GC rich regions, especially within the 5′ and 3′ UTRs [30], which largely share with the regions susceptible to the formation of R-loops [31, 32]. These data suggested that R-loop-associated DNA damage response may contribute to eccDNA generation. Similarly, DNA-damaging agents, such as hydroxyurea (HU) [33], have helped provide evidence for the role of DNA damage repair (DDR) in extrachromosomal circular DNA production. Specific proteins in DDR, for example, DNA-PKcs (a central player in nonhomologous end joining (NHEJ)) [34] and MSH3 (involved in mismatch repair (MMR)), are necessary for the formation of extrachromosomal circular DNA [35]. In support of these findings, Dillon et al. [31] noted that deletion of the DNA repair protein MSH3 resulted in a dramatic decrease in the amount of circular DNA in human ovarian and prostate cancer cell lines. In addition, immunoblot analysis has shown that depletion of DNA-PKcs by shRNA or inhibitors causes the decreased amplification of *dihydrofolate reductase (DHFR)* on ecDNA and the elimination of ecDNA in the methotrexate (MTX)-resistant colon cancer cell line HT-29 [36]. This finding supports the notion that NHEJ is important for the formation of ecDNA. Moreover, the depletion of the Ku70/80 heterodimer significantly decreases the level of circular extrachromosomal DNA and inhibits the proliferation of human osteosarcoma SAOS2 cells [37]. Another DDR pathway, homologous recombination (HR), is also involved in extrachromosomal gene amplification. The attenuation of HR activity by shRNA-mediated BRCA1 depletion reduces the amount of extrachromosomal circular DNA and circular DNA-amplified *DHFR* in MTX-resistant colon cancer cells [38]. However, in contrast to these findings [38], defects in the HR protein RAD54 were shown to induce a markedly increased number of subpopulations containing extrachromosomal circular DNA in MTX-resistant subclones from HeLa cervical cancer cells [39]. This contradiction might be due to the lack of sufficient repetitive elements in the HeLa cell genome for the recombination pathway to loop out extrachromosomal circular DNA. To some extent, additional compensatory mechanisms, such as NHEJ, could contribute to the formation of extrachromosomal circular DNA after RAD54 inhibition, but this requires further exploration.

Previous reports support the general idea that extrachromosomal circular DNA might originate from either repeat or nonrepeat sequences within a genome. A survey of tandem repeat sequences in eccDNA found that chromosomal tandem repeats were excessively represented in eccDNA from the HeLa and SW620 cancer cell lines [40]. Moreover, the transcription of tandem repeat sequences can potentially lead to the accumulation of eccDNA via intrachromosomal HR between tandem repeats, such as extrachromosomal rDNA circles (ERCs), ultimately leading to genome instability [41, 42]. However, in HeLa S3 cells, nearly half of the eccDNA fragments were entirely composed of nonrepetitive DNA sequences [43]. In human lymphoblastoid cell lines, an analysis of microDNA features showed that abundant microDNA is mainly derived from nonrepetitive genomic regions [30], supporting the contribution of nonhomologous recombination in circular DNA production. Thus, these unique sequences could serve as valuable resources for the generation of circular DNA. Moreover, as dysfunction of DNA repair pathways causes the accumulation of DNA damage involved in tumorigenesis [44], this underlying knowledge provides novel insights into the formation of circular DNA by genomic sequences and might present promising genomic strategies to restrict tumor progression by influencing circular DNA abundance.

As mentioned above, although DNA damage responses are now available to generate ecDNA, the specific mechanisms that control DM generation in cancers are still not fully understood (Fig. 3). Previously, chromothripsis was proven to explain the origin of DMs in some cancer cells [45]. Chromothripsis is a single-step catastrophic event that drives local chromosome breakage and end-to-end chromosomal fusion and serves as the underlying driving force linked to DMs. For example, the genesis of complex DMs in several cancer types, including glioblastoma (GBM) and small cell lung cancer, has been explained by the telomeric fusion of chromosomes broken by chromothripsis [46, 47]. Recently, another model for DM genesis was proposed, named the episome model [48]. In support of this idea, a growing body of research evaluates the fine structure of *c-Myc*-containing DMs in different types of cancer cells [49, 50]. These findings consistently identify a stepwise recombination process, starting from single-chromosome amplified episomes, for the generation of *c-Myc*-containing DMs. Moreover, different sizes and architectures of *c-Myc* amplicons can coexist within the same cell population, providing evidence of DM heterogeneity. Intriguingly, several fusion genes or circular RNA of *plasmacytoma variant translocation 1 (PVT1)* can occur frequently in cells with *c-Myc* amplicons, further highlighting the role of PVT1 as a breakpoint hotspot for chromosomal breaks during *c-Myc* amplification and synergistically contributing to tumorigenesis [51]. Other mechanisms of DM generation have also been reported, such as breakage-fusion-bridge (BFB) cycles [52, 53], although their detailed roles have yet to be determined. In brief, during mitosis, the BFB cycle involves anaphase bridge formation due to the presence of dicentric chromosomes, followed by bridge breakage, which generates a variety of chromosomal aberrations, including DMs.

### The heterogeneity of extrachromosomal circular DNA in cancer

Owing to different genetic backgrounds and tissue types, these circular DNA molecules have broad heterogeneity in size [54], including small circularized DNA entities, later referred to as eccDNA, and larger ecDNA (Table 1). Subsequent studies revealed that the quantity of circular DNA has been widely reported for different types of cancer and might have the potential to amplify and drive intratumor genomic heterogeneity. Because of the advanced molecular and functional profiles, eccDNA/ecDNA elements are now understood to be crucial to oncogenetic plasticity [47, 55] and may be associated with poor prognosis [56]. More importantly, the biological roles of DNA circularization in the regulation of cancer-associated genes facilitate the more effective adaptation of cancer cells to variable environmental stresses, propagating tumor pathologies and drug resistance [57].

**Table 1:** The classification of extrachromosomal circular DNA in cancer

Name	Size range	Replication	Function
SpcDNA	Several hundred bp	Self-replicate	Contributing to telomere homeostasis
T-circles/c-circles	0.3 to 30 Kb	Self-replicate	Serving as templates for telomere elongation
MicroDNA	100 to 400 bp	Self-replicate	Regulating microRNAs
ERCs	19.3 to 40.4 Kb	Self-replicate	Serving as templates for ribosomal RNA transcription
DMs	A few to several Mb	Self-replicate	Leading to oncogene amplification and overexpression

#### SpcDNA

Small polydisperse circular DNA (spcDNA) of several hundred base pairs (bp) is one kind of eccDNA element in cells, and it shares homology with chromosomal DNA sequences [58]. In general, several groups have detected double-stranded spcDNA in different organisms under physiological and pathological conditions [9, 59]. Using southern hybridization with telomeric probes, Regev and colleagues verified the telomeric repeat sequences in the structures of double-stranded spcDNA and named these DNA molecules tel-spcDNA [60]. However, an interesting question is whether and how spcDNA formation directly contributes to telomere homeostasis. Subsequent studies offer a possible clue that spcDNA in telomerase-negative cancer cells can effectively maintain telomere length via the telomerase-independent telomere-restitution pathway [61]. Notably, the analysis of circle integration showed that spcDNA also causes genomic rearrangements by inserting and connecting a region proximal to the *TERT* gene, finally leading to enhanced *TERT* expression in neuroblastoma cells [17]. These data revealed that spcDNA is actively involved in the telomere dynamics that occur during tumorigenesis. Therefore, the function of spcDNA in telomere regulation might have potential roles in the surveillance of tumor progression. In addition, spcDNA levels are more than 3-fold higher in cancer cells than in normal cells [62] and are further enhanced by treatment with carcinogen [60]. Other reports have shown that the elevated amount of spcDNA is considerably connected with proliferating melanoma cells [63]. Furthermore, spcDNA has been identified to serve as an enhancer of genetic instability, contributing to cancer development and progression [14]. Thus, these observations highlight the importance of spcDNA existence in malignant pathology.

#### Extrachromosomal circles of telomeric DNA

The mechanism, alternative lengthening of telomeres (ALT), has been proven to be responsible for telomere maintenance by a telomerase-independent mechanism and to affect tumor biology [64, 65]. ALT, a noncanonical mechanism of telomere maintenance, is frequently developed by cancer cells with non-functional telomerase [66]. Currently, accumulating evidence suggests that the ALT pathway can be examined by the presence of extrachromosomal circles of telomeric DNA, double-stranded t-circles, or single-stranded c-circles [67–69]. In particular, unlike other categories of extrachromosomal circular DNA species, t-circles/c-circles are highly specific for the ALT mechanism and can be used as promising biomarkers for the diagnosis and management of ALT-positive tumors [70]. Electron microscopy assays show that these telomeric DNA circles, ranging in size from 0.3 to 30 Kb, are conserved structures in yeast and human ALT cells [71]. In principle, t-circles/c-circles can serve as templates for telomere elongation by a rolling-circle mechanism, thereby contributing to telomere maintenance and cell proliferation in cancer cells [72–74]. Accordingly, the c-circle concentration in B-chronic lymphocytic leukemia is increased compared to that in normal B cells [75]. The presence of c-circles dramatically correlated with enhanced telomeric DNA content and increased survival in malignant glioma samples [76]. Likewise, by characterizing telomere structures in cells and tissues from patients with high-risk neuroblastoma, Yu et al. [77] found that neuroblastoma cells harbor high levels of t-circles, which may be due to active “telomere trimming”, a pathway that entails recombination excision of telomere repeats. Moreover, the extreme trimming activity in neuroblastoma cells results in rapid telomere deletion and increased t-circles, dramatically promoting extensive proliferation [78]. Given their important function in cancer progression, t-circles/c-circles have attracted considerable attention due to their precise diagnostic and therapeutic potential for cancers with the ALT phenotype.

Insights into the t-circle/c-circle formation mechanisms remain limited; to date, several DNA damage-associated proteins, such as XRCC3 [79], NBS1 [64], and Ku70/80 [37], have been strongly implicated in this process [80]. Moreover, because of the lack of the ability to initiate replication, t-circles/c-circles are mainly generated from recombination rather than heritably amplified episomes. The production of t-circles/c-circles using homologous recombination can be obviously inhibited by RAD52 deletion [81, 82]. ATRX loss leads to multiple phenotypic features of ALT in two high-grade glioma cell lines, U-251 and UW479, including c-circle formation [83]. Knockdown of the Ku70/80 heterodimer by shRNAs reduces the levels of t-circles and activates the p53 pathway, ultimately resulting in significantly decreased cell growth in SaOS2 osteosarcoma cells [37]. However, another study showed that while XRCC3 and NBS1 are required for t-circle production, knocking down these two factors does not affect cell proliferation and telomere maintenance [84]. These unexpected findings suggest that in the absence of t-circles/c-circles, there may be some subtypes of cancer cells undergoing additional telomere maintenance pathways, for example, overexpression of telomerase components [17, 85]. Further studies are needed to better understand the mechanistic details of t-circles/c-circles on telomere maintenance and cell growth in ALT cancer cells. In addition to these DNA repair-associated proteins, whether other factors are responsible for t-circle/c-circle formation in different cancers is an interesting question for future investigation. To prove this hypothesis, Touzot et al. [86] demonstrated that the regulator of telomere elongation helicase 1 (RTEL1) is required for telomere stability and thereby prevents rapid telomere deletion and t-circle formation. Moreover, high levels of TERT and telomerase activity can suppress the production of c-circles in ALT-positive cells from brain tumors [87] and promyelocytic leukemia [88]. In addition, deficiency of several telomere-associated factors, such as the CST complex (CTC1-STN1-TEN1) [89], telomeric repeat-containing RNA [90] or homeobox containing protein 1 [91], has been found to significantly increase telomeric DNA damage and diminish the abundance of t-circles/c-circles, thereby impairing cell proliferation in U2OS osteosarcoma cells.

#### MicroDNA

MicroDNA, with an average of 100 to 400 bp, is derived from nonrepetitive genomic regions with high gene density and has been suggested to be the most prevalent subtype within the eccDNA [92, 93]. Of note, microDNA is widespread in all mammalian tissue types investigated to date and might become a promising target for future cancer research. In support of this hypothesis, microDNA from tumor cells has been shown to be substantially larger than that from normal somatic cells [21]. A recent study addressing the microDNA features in lymphoblastoid cells proposed that MTX-treated cells also display significantly longer and higher numbers of microDNAs when compared to nontreated cells [30]. Moreover, the profiling of microDNA in circulation shows that the abundance of cell-free microDNA is highly consistent with tumor burden in lung cancer patients [94]. Additionally, because they contain microRNA coding sequences, some microDNA from exon regions within the genome expresses functional regulatory microRNAs, such as hsa-let-7a, and represents a novel RNA interference (RNAi)-like mechanism that changes gene expression in cancer cells [8]. Considering such evidence, microDNA in tumor cells and circulation might both serve as attractive biomarkers for monitoring cancer progression and therapeutic efficacy.

#### Extrachromosomal rDNA circles

Extrachromosomal rDNA circles ERCs, with an average of 19.3 to 40.4 Kb, can serve as a template for ribosomal RNA transcription and are the most abundant eccDNA molecule in healthy tissue [95]. Studies have demonstrated that accumulated ERCs in cells are formed from the repetitive rDNA locus in the genome through both random and environmentally stimulated HR processes [57, 96]. Moreover, ERCs have an autonomously replicating sequence and are able to self-replicate [97]. Changes in the copy number of ERCs have been proven to be associated with functional gene variations across the genome and are also particularly associated with DNA damage sensitivity and cancer [40]. In response to DNA damage, increased ERCs have been thought to be a potential contributor to several age-related diseases, including cancers [98]. All these observations preliminarily suggest the probable roles of ERCs in cancer biology, although their detailed function remains largely unknown. Further experiments will be necessary to prove their action in tumorigenesis.

#### Double minutes

DMs are currently well-characterized ecDNA, and these molecules, which range in size from a few to several Mb, accumulate in tumor cells; this accumulation is the cytogenetic hallmark of the extrachromosomal genomic amplification of oncogenes [99, 100]. In cells, all DMs have been demonstrated to be closely coupled. It is well known that DMs, similar to intrachromosomal homogeneously stained regions, contain copies of an amplified DNA segment (the amplicon), leading to gene amplification and overexpression [101]. DMs are autonomously replicating circular chromatin bodies that lack recognizable centromeres and telomeres and are frequently identified in cytogenetic examinations of metaphase chromosomes in human solid cancer cells [102]. Unexpectedly, even though DMs are rare in myeloid neoplasms [103], they are generally associated with myelodysplasia and therapy-related side effects, also resulting in poor prognosis in patients with leukemia [104–106]. Moreover, studies have demonstrated that DMs can evolve toward ring chromosomes that are stabilized by neocentromeres (ectopic centromeres), providing an evolutionary advantage to leukemia cells [50]. Molecularly, these circular DMs provide a template for the amplification and upregulation of oncogenes in a rolling-circle amplification mechanism. To date, multiple oncogenes have been identified on DMs, especially *c-Myc*, *MYCN*, and *EGFR* [107]. Moreover, the study by Fan et al. proposed that the occurrence frequency of DMs in malignant cancers is much higher than that in benign cancers or noncancerous tissues [108], suggesting that those DMs can be exploited as reliable biomarkers of tumor progression.

In addition, the copy number of these circular elements can be significantly altered in response to environmental changes. One recent study showed that gemcitabine, a nucleoside analog used as chemotherapy, is effective in decreasing DMs in the ovarian cancer cell line UACC-1598 [109]. The authors propose that elimination of DMs, as well as the amplified genes in DMs, seems to be quite important because it decreases the malignant phenotype of cancer cells. Similarly, several DNA-damaging agents, including cisplatin, doxorubicin, and HU, significantly decrease the amplified *MYCN* in DMs from neuroblastoma cancer cells and thereby reduce tumorigenicity [110]. More importantly, if DMs can be explored and characterized accurately with cytogenetic methods [111], it might help the researcher evaluate the effectiveness of anticancer drugs on the subclones of cancer cells that contain DMs.

### Extrachromosomal circular DNA-based oncogene overexpression

The chimeric circularization and amplification of circular DNA is very common and typically has a profound impact on the enhanced expression of oncogenes. However, it is unclear whether circularization itself or subsequent copy number amplification induces upregulation of the various oncogenes. To address this question, a recent investigation conducted by Koche and colleagues [17] demonstrated that the majority of oncogene amplification as determined by WGS seemed to coincide with circular DNA amplification in neuroblastoma. Likewise, by applying haplotype phasing analysis, the authors found that circular DNA is mainly derived from amplified alleles. Together, these findings confirmed that extrachromosomal DNA circularization served as an important driver of the high level of oncogenic amplification. These observations further suggest the use of circular DNA as a re-emerging target for the development of novel anticancer therapeutic strategies.

Unexpectedly, however, some oncogenes with variable copy numbers contained in circular DNA were not altered in neuroblastoma cells [17], indicating that extrachromosomal circular DNA might be required but insufficient alone to increase gene amplification. Based on this striking phenomenon, we hypothesized that circular DNA might have additional important functions in tumor pathogenesis. To clarify this issue theoretically, subsequent quantitative assessment of the chromatin state found that ecDNA could also strongly promote the transcription of full-length or truncated oncogenes by integrating back into the active chromatin with their intact domain structure [4, 112].

To date, progress in genetic research has revealed additional roles of circular DNA beyond its function in promoting oncogene amplification or transcription. This circular DNA may also drive oncogenic remodeling in human cancers, resulting in significantly adverse clinical prognoses [113]. Owing to a lack of integrative and highly sensitive strategies to characterize circular DNA in cells [114], such genomic rearrangements have often remained relatively underestimated or undetected by previous WGS methods. By applying a sensitive analysis algorithm that combines WGS datasets with the complementary sequencing of circular DNA (Circle-seq), Koche et al. [17] generated a landscape of tumor-specific circular DNA molecules in human neuroblastoma cells. The authors concluded circle-derived somatic rearrangements followed by reintegration of the circular DNA molecules into the linear genome. The authors also reported that most of the somatic chromosomal rearrangements were from the regions of DNA circularization, providing a scientific basis for the proposed hypotheses that circular DNA plays a profound role in genome remodeling. One can imagine that such a widespread reintegration phenomenon might have important effects on chromosomal gene aberrations, especially in the overexpression of oncogenes and the activation of oncogenic kinase signaling [115].

### Extrachromosomal circular DNA-linked drug resistance

Today, multiple therapeutic methods have been widely used for cancer patients in clinical practice, including standard chemotherapy and targeted therapy. However, drug resistance is an almost universal challenge in cancer therapy, ultimately leading to tumor recurrence and treatment-related morbidity [116]. The cellular and molecular mechanisms of therapy resistance are quite complicated and involve multiple factors and signaling pathways [117]. For example, extrachromosomal amplified DNA and its regulatory capacity have been determined to play key roles in driving tumor heterogeneity and regulating therapy resistance in various cancers [118, 119], including colon cancer and GBM (Table 2). Defining the extrachromosomal DNA that drives the amplification of drug-related genes would aid in providing novel therapeutic perspectives to reverse resistance and ameliorate outcomes. Extrachromosomal circular DNA could promote the amplification of drug resistance-associated genes, and it is a powerful driver of intercellular genetic heterogeneity [120]. The classical MTX resistance gene *DHFR* was proven to amplify primarily in the form of DMs, contributing to tumor progression and the development of MTX resistance in human colon cancer cells. The dramatic impairment of HR [38] and NHEJ [36] repair activity could suppress the level of DHFR-amplified DMs and considerably revert the sensitivity of HT-29 cells to MTX but not affect intrachromosomal *DHFR* amplification. These findings support previous conclusions that targeting *DHFR* in cancer cells harboring amplified *DHFR*-containing circular DNA has attractive therapeutic potential [121]. However, substantial work is urgently needed to investigate the factors that determine the sensitivity of *DHFR*-amplified cancers in anti-DHFR treatment.

**Table 2:** Summary for the extrachromosomal circular DNA-linked drug resistance

Genes	Drugs	Function	Cancers	Refs
*DHFR*	MTX	Elimination of *DHFR* on DMs increases MTX sensitivity	Colon cancer	[36]
*DHFR*	MTX	Elimination of *DHFR* on DMs increases MTX sensitivity	Colon cancer	[38]
*DHFR*	MTX	Amplification of DM-form *DHFR* promotes MTX resistance	Cervical cancer	[39]
*DHFR*	MTX	X-ray induces MTX resistance due to DM-form amplified *DHFR*	Breast Cancer	[121]
*EGFRvIII*	Erlotinib	Reducing *EGFRvIII*-bearing extrachromosomal DNA resultes in erlotinib resistance	Glioblastoma	[128]
*MDM2*	Erlotinib	Amplification of DM-form *MDM2* promotes erlotinib resistance	Glioblastoma	[128]
*HER2*	Trastuzumab	Loss of DM-form *HER2* has no effect on trastuzumab resistance	Breast Cancer	[129]
*c-Myc*	HU and retinoic acid	Reducing *c-Myc*-bearing DMs enhances therapeutic sensitivity	Leukemia	[134]
*MYCN*	HU	Elimination of *MYCN* on DMs increases HU sensitivity	Neuroblastoma	[135]
*c-Myc*	HU	Reducing *c-Myc*-bearing DMs enhances HU sensitivity	Colon cancer	(136)
*MDR1*	HU	Loss of DM-form *MDR1* promotes HU sensitivity	Oral squamous cell carcinoma	[137]
*CA125*	HU	Low levels of DM-form *CA123* after HU treatment	Ovarian cancer	[138]

In addition, there is evidence that regions of genomic rearrangement, such as genomic focal amplification sites in human cancers, might be closely related to strong gain-of-function mutations in oncogenes, contributing to the development of drug resistance in cancer therapy [122]. Circular DNA elements act as the cytogenetic hallmarks of genomic focal amplification in cancer cells [123], and the copy number of these elements with oncogenic mutations is altered in response to environmental changes [107]. An example of this is in nervous system neoplasms, including GBM and low-grade glioma, where the receptor tyrosine kinases (RTKs) are frequently mutated and commonly give rise to the driver variant [124]. In particular, the activating mutations of *EGFR* [125] and *PDGF receptor a (PDGFRa)* [126], two members of the RTK family, have usually been thought to reside primarily on DM structures. These extrachromosomal mutations are the driving factors that contribute to therapy resistance by increasing tumor heterogeneity. Studies have also indicated that although the constitutively active mutant of EGFR, *EGFR variant III (EGFRvIII)*, plays critical pro-survival roles in GBM pathogenesis, and it also makes cancer cells more sensitive to EGFR inhibitors [127]. Therefore, resistance to EGFR-targeted therapy might develop if extrachromosomal mutations are eliminated. In recent years, several empirical studies have been conducted to address this issue. After treatment with the EGFR inhibitor erlotinib, *EGFRvIII*-bearing extrachromosomal DNA elements within GBM cells were markedly reduced, resulting in resistance to anti-EGFR therapeutics. However, after withdrawal of the drug, the reemergence of clonal *EGFRvIII* mutations on DMs effectively resensitized cancer cells to EGFR inhibitors, which could then induce cell death [128]. Unexpectedly, in some cancer cell subclones, resistance to anti-EGFR therapy might not be entirely mediated by *EGFRvIII*-positive DMs; rather, resistance might occur through other compensatory mechanisms, such as the potential oncogenic roles of extrachromosomal *MDM2* amplification [128]. In addition, Vicario et al. [129] analyzed the amplification patterns of *HER2* (another member of the RTK family) in response to different anti-HER2 therapies. Although the acquisition of resistance in HER2-positive breast cancer cells is often concomitant with HER2 protein loss, the copy number of *HER2*-positive DMs did not obviously change after treatment with the HER2 inhibitor trastuzumab. These data indicate that HER2 elimination-mediated resistance might not be due to the loss of *HER2*-containing circular DNA. Thus, future work is needed to evaluate the compensatory mechanisms that enable cancer cells with or without RTK-positive extrachromosomal DNA to continue to proliferate during anti-RTK treatment. By elucidating these dynamic regulatory mechanisms in detail, we will gain more comprehensive insight into therapy resistance, and this understanding could support the future development of novel therapeutic approaches.

**Fig. 4:**
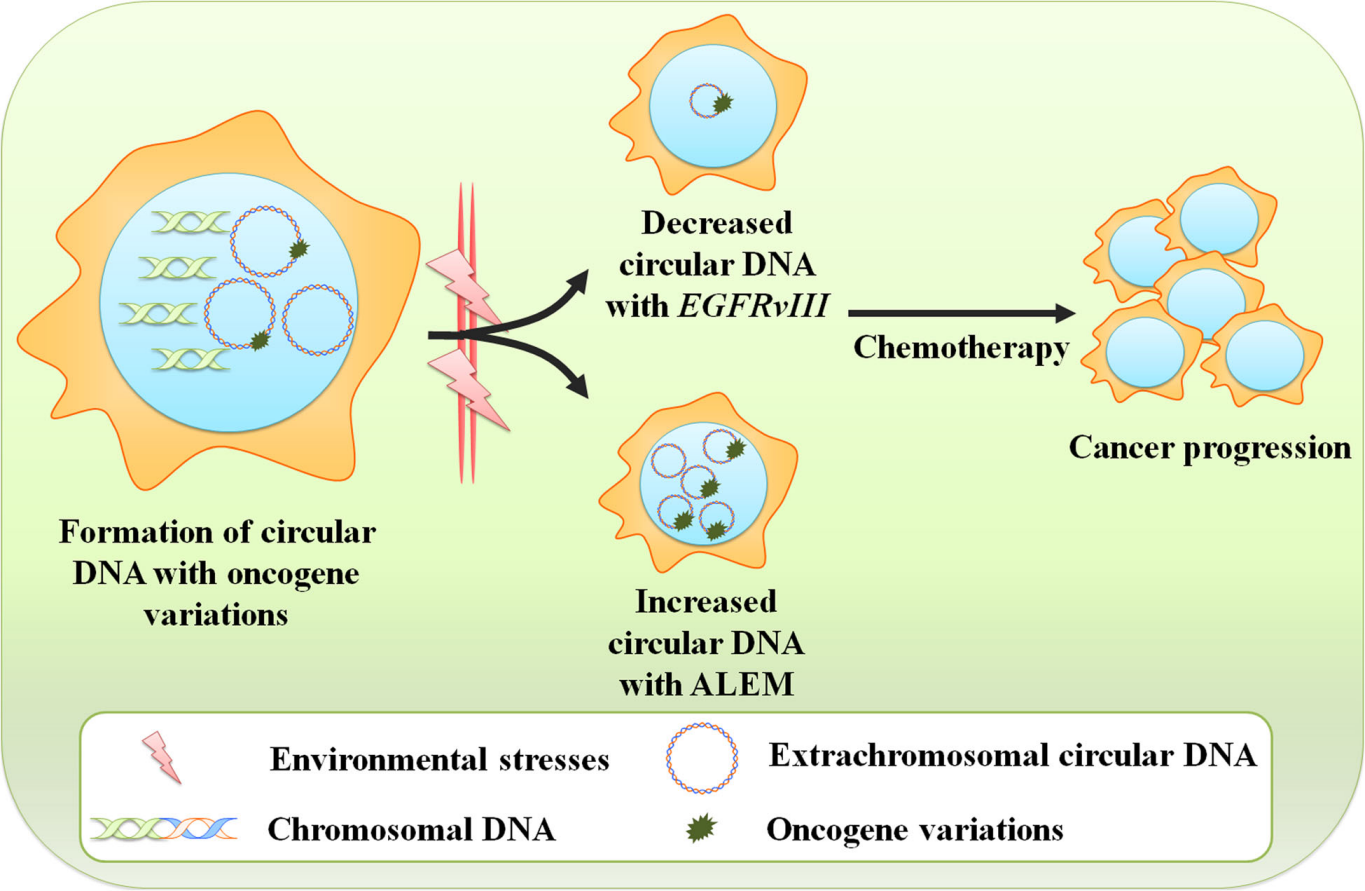
Roles of extrachromosomal circular DNA with oncogene variation in therapeutic response. After the formation of extrachromosomal circular DNA, the amplified circular DNA elements are prone to pass on oncogene variations upon amplification, such as *EGFRvIII* and ALEM. In response to environmental changes, cancer cells might accordingly increase or decrease the copy number of circular DNA elements containing oncogene variations, leading to anticancer therapy resistance

Other extrachromosomal driver mutations could also occur during the amplification of DMs, and this might be critical for tumor heterogeneity and therapy resistance. By applying exome sequencing to seven GBM tissue samples, Nikolaev et al. [130] identified a novel class of extrachromosomal gain-of-function mutations mediated by focal amplification. These amplification-linked extrachromosomal mutations (ALEMs) were found in multiple members of the RTK family, such as *EGFR* and *c-KIT*. Because of the unequal segregation of DMs between daughter cells during cell division, some daughter clones may inherit a higher copy number of DMs carrying driver ALEMs and thus obtain a growth advantage (Fig. 4). This unequal segregation effect, also known as “hitchhiking,” might be widespread across different types of cancers [131]. Interestingly, based on the “hitchhiking” mechanisms, DM-originating ALEMs could enable cancer cells to undergo rapid adaptation to environmental stresses, thereby acquiring resistance to anticancer therapies [130]. Overall, these studies emphasize that extrachromosomal DNA contributes to therapy resistance in cancer cells in multiple ways. These mechanisms could be exploited in further research and future clinical trials.

### Clinical utility

Tumor-specific features of extrachromosomal circular DNA elements could be important for the development and progression of malignancies and for therapy resistance [8]. It is therefore important to explore the extrachromosomal DNA profiles of patients with advanced cancers in clinic (Table 3). It has been proposed that extrachromosomal circular DNA is the dominant manifestation of amplified drug-resistance genes and oncogenes in patient-derived tumor tissues and can be identified in multiple types of cancers [132, 133]. Therapeutic strategies that could reduce extrachromosomal DNA elements in cancer cells would likely downregulate the gene expression that contributes to aggressive phenotypes and drug resistance in advanced cancers. Indeed, increasing *in vitro* and *in vivo* preclinical evidence has demonstrated that the loss of DMs, accompanied by reduced levels of DM-form oncogenes (*c-Myc*, *MYCN*, *MDR1*, etc.), can be achieved upon drug treatment and further enhance the therapeutic sensitivity [134–136]. Likewise, the results from independent studies have found that elimination of the amplified drug resistance genes on extrachromosomal circular DNA would be feasible therapeutic strategies to make the cells more sensitive to chemotherapy drugs, such as HU and MTX [137, 138]. Based on these preclinical findings, Raymond et al. recently performed a single-arm study of 16 patients with advanced ovarian carcinomas and found that orally administered HU at a noncytotoxic dose decreased the DM copy number in cancer cells and improved the prognosis dramatically [139]. Thus, noncytotoxic strategies that eliminate DMs could be helpful in personalizing treatment decisions in cancer patients. Although these findings are interesting, future clinical trials to verify the efficacy of noncytotoxic strategies are now warranted for ovarian cancer and other types of cancers with frequently amplified DMs.

**Table 3.** The potential clinical utility of extrachromosomal circular DNA elements in cancer patients

Clinical applications	Refs
• Change the levels of extrachromosomal oncogenes influence the treatment efficacy	
(i) Reemergence of clonal *EGFRvIII* mutations on DMs resensitizes cancer cells to anti-EGFR inhibitors	[128]
(ii) Treatment with chemotherapeutic drugs accelerates the loss of extrachromosomally amplified genes	[137]
(iii) Elimination of extrachromosomal genes increases the drug sensitivity	[133]
• Extrachromosomal DNA serves as the ideal biomarkers for clinical monitoring	
(i) Cancer-derived EVs carrying extrachromosomal DNA can transfer the fragments of some oncogenes and trigger malignant progression	[141]
(ii) Circular DNA elements can be detected in the blood-based screening of plasma from cancer patients as a component of liquid biopsy	[150]
(iii) Significantly decreased extrachromosomal circular DNA in circulation following tumor resection in cancer patients	[94]

In addition, it has recently been proven that some extracellular DNA is enriched in cancer-derived extracellular vesicles (EVs), further contributing to their stability [140]. In human body fluids, these cancer-derived EVs carrying circular extrachromosomal DNA has been suggested to trigger a multiplicity of physiological and pathological responses [141]. These EVs can also functionally transfer fragments of some genomic oncogenes, such as *H-ras* [142] and *K-ras* [143], and result in the oncogenic transformation of epithelial cells. It was further observed that EVs containing extrachromosomal DNA can activate the paracrine signaling with neighboring cells, ultimately contributing to malignant biological behaviors and modulation of anti-cancer therapy [144, 145]. A recent work conducted by Read et al. showed that EVs derived from prostate cancer cell lines transferred *EGFR* and *androgen receptor (AR)* into extracellular space, which enhances the malignant phenotypes of neighboring cells [146]. Of course, this comprehensive knowledge provides important and new insights into the utilization of extrachromosomal circular DNA in EVs as biomarkers for cancer research and treatment.

In light of their important roles in oncogene plasticity and drug resistance, circular DNA elements can be detected in the blood-based screening of plasma from cancer patients as a component of liquid biopsy [147, 148]. Especially, several studies have indicated the potential application of extrachromosomal DNA elements in body fluids as candidate biomarkers for the diagnosis and monitoring of several disorders, such as cancers [149]. Because of their extraordinary structural stability than linear DNA, circular DNA in the blood might serve as an ideal source of potential biomarker exploration [150]. In support of their value as clinical monitoring tools, several recent reviews highlighted that the length and abundance of circular DNA elements have been more prominent in the cancer patients compared with the healthy donors [9, 133]. The analysis of pre- and post-surgery plasma from cancer patients revealed significantly decreased levels of eccDNA in blood circulation following tumor resection [35]. Accordingly, Kumar’s group recently identified the microDNA released from cancer cells in serum and plasma from cancer patients and characterized these unique molecular profiles after anti-cancer treatment [94]. Likewise, the abundance of DMs in peripheral blood lymphocytes has been demonstrated to function as an independent risk factor for patients with lung cancer [151]. Sequencing analysis of eccDNA profiles revealed thousands of unique circular DNAs in the circulatory system, with a highly heterogeneous molecular mass profile; the size distribution ranged from several base pairs to several kilobase pairs [150]. Thus, the discovery of cancer-associated circular DNA in the circulation provides a scientific basis for further investigations of the use of these DNA elements as biomarkers for precision cancer therapies. The real-time monitoring of extrachromosomal circular DNA in circulating blood has provided novel insights into cancer pathology. The clinical application of detecting circular DNA in liquid biopsies to identify tumorigenesis and progression is attractive; however, some problematic issues remain. The most important is a lack of reliable methods for easily quantifying the abundance of circular DNA elements in the blood [152].

### Toolbox for extrachromosomal circular DNA analysis

While circular extrachromosomal DNA has been able to be determined for some time, higher-resolution imaging and cytogenetic methods have only recently been developed to evaluate these molecules. Advances in sequencing technologies have allowed for the genome-scale screening and mapping of circular DNA from human cancer cells [153]. Based on short-read sequencing and connecting amplified DNA segments, some computational analysis tools have been developed to explore the profiles of circular DNA signals in tumor samples in an unsupervised manner [154], such as AmpliconArchitect [155]. Defining eccDNAs using AmpliconArchitect showed that oncogenes mainly amplify eccDNA elements and are surprisingly preserved during cancer progression, which has been shown in previous reports [11]. However, these short-read maps could not unambiguously align the long-range duplications crossing the circular structure. As a consequence, the characterization and functional profiles of circular DNA elements might have been drastically underestimated in cancer patients. To effectively solve these problems, the detection systems are expected to be improved in subsequent studies. Recently, several improved tools with high precision, such as AmpliconReconstructor [131] and Circle-Map [156], were developed to better map the physical structure of long contiguous reads. Based on the BioNano technology platform, AmpliconReconstructor was used to provide more conclusive evidence that oncogenes carrying ecDNAs produced substantially more transcripts in multiple cancer cells and tissue types. Today, other higher-resolution technologies are being developed to improve the resolution of circular DNA from high-throughput DNA sequencing data. For example, a higher-resolution image analysis algorithm, named ECdetect, has been developed and is used to effectively quantify ecDNAs from DAPI-stained metaphases in an unbiased and highly accurate fashion [11, 55]. Taken together, these effective tools will permit a deeper understanding of the characteristics of extrachromosomal DNA particles in cancer pathology and their association with clinical features.

It is also essential to expand the cytogenetic toolbox to explore the function of key components with respect to extrachromosomal DNA particles. For a long time, fluorescence in situ hybridization (FISH)-based approaches have been used to resolve amplified oncogenes linked with cancer development. However, FISH probes have been unable to discriminate the location of specific genes on chromosomal and extrachromosomal DNA molecules [157]. A more advanced cell-imaging technology, ecSeg [158], has been introduced to accurately quantify oncogene amplification from ecDNA at the single-cell level. Moreover, the application of genome editing techniques, such as CRISPR-Cas [159, 160], could be leveraged to advance our understanding of the basic biologic functions of these molecules, especially in circular DNA-driven oncogene amplification and drug resistance. Moreover, as delineated above, such methods could help researchers discover agents that target extrachromosomal circular DNA elements.

## Conclusions

As mentioned above, cancer-associated genes can be amplified in circular extrachromosomal DNA elements. In particular, because of the unequal segregation into daughter cells, the amplification of eccDNA/ecDNA elements could be a primary mechanism by which oncogenes rapidly reach higher transcript levels and copy numbers than in intrachromosomal amplification. There is growing evidence supporting the functional importance of circular DNA in promoting genetic heterogeneity and accelerating the progression of human cancer pathologies. Moreover, the amplification of circular DNA elements containing oncogenes (such as *EGFR* and *c-Myc*) could help cancer cells adapt more effectively to variable environmental stress by acquiring fate-enhancing advantages. Thus, some subclones would express higher levels of oncogenes, leading to cancers that become more aggressive, have poorer prognoses, and are more difficult to treat clinically over time. Accordingly, understanding the underlying molecular mechanisms of tumor heterogeneity, including oncogene amplification from circular extrachromosomal DNA, might help to develop remarkable potential therapeutic strategies that either prevent cancer progression or overcome therapy resistance. Such important scientific topics are currently being addressed by pioneering novel work on these issues.

## Data Availability

Please contact the corresponding author for all data requests.

## Abbreviations

WGS: Whole-genome sequencing
EGFR: Epidermal growth factor receptor
EGFRvIII: EGFR variant III
MDM2: Mouse double minute 2
CCND1: Cyclin D1
DCLK1: Doublecortin-like kinase 1
TERT: Telomerase reverse transcriptase
HU: Hydroxyurea
DDR: DNA damage repair
NHEJ: Nonhomologous end joining
DHFR: Dihydrofolate reductase
MTX: Methotrexate
HR: Homologous recombination;
rDNA: Ribosomal DNA
Circle-seq: Complementary sequencing of circular DNA
PDGFRa: PDGF receptor a
ALEMs: Amplification-linked extrachromosomal mutations
FISH: Fluorescence in situ hybridization
SpcDNA: Small polydisperse circular DNA
ERCs: Extrachromosomal rDNA circles
DMs: Double minutes
PVT1: Plasmacytoma variant translocation 1
BFB: Breakage-fusion-bridge
AR: Androgen receptor
EVs: Extracellular vesicles
